# Habitat suitability of biocrust communities in a cold desert ecosystem

**DOI:** 10.1002/ece3.11649

**Published:** 2024-06-28

**Authors:** Sarah N. Power, Valerie A. Thomas, Mark R. Salvatore, John E. Barrett

**Affiliations:** ^1^ Department of Biological Sciences Virginia Polytechnic Institute and State University Blacksburg Virginia USA; ^2^ Department of Forest Resources and Environmental Conservation Virginia Polytechnic Institute and State University Blacksburg Virginia USA; ^3^ Department of Astronomy & Planetary Science Northern Arizona University Flagstaff Arizona USA

**Keywords:** biocrust, carbon, dryland, habitat suitability, remote sensing, soil ecology

## Abstract

Drylands are unique among terrestrial ecosystems in that they have a significant proportion of primary production facilitated by non‐vascular plants such as colonial cyanobacteria, moss, and lichens, i.e., biocrusts, which occur on and in the surface soil. Biocrusts inhabit all continents, including Antarctica, an increasingly dynamic continent on the precipice of change. Here, we describe in‐situ field surveying and sampling, remote sensing, and modeling approaches to assess the habitat suitability of biocrusts in the Lake Fryxell basin of Taylor Valley, Antarctica, which is the main site of the McMurdo Dry Valleys Long‐Term Ecological Research Program. Soils suitable for the development of biocrusts are typically wetter, less alkaline, and less saline compared to unvegetated soils. Using random forest models, we show that gravimetric water content, electrical conductivity, and snow frequency are the top predictors of biocrust presence and biomass. Areas most suitable for the growth of dense biocrusts are soils associated with seasonal snow patches. Using geospatial data to extrapolate our habitat suitability model to the whole basin predicts that biocrusts are present in 2.7 × 10^5^ m^2^ and contain 11–72 Mg of aboveground carbon, based on the 90% probability of occurrence. Our study illustrates the synergistic effect of combining field and remote sensing data for understanding the distribution and biomass of biocrusts, a foundational community in the carbon balance of this region. Extreme weather events and changing climate conditions in this region, especially those influencing snow accumulation and persistence, could have significant effects on the future distribution and abundance of biocrusts and therefore soil organic carbon storage in the McMurdo Dry Valleys.

## Graphical abstract

Biocrusts are dominant primary producer communities inhabiting the McMurdo Dry Valleys of Antarctica. We found that gravimetric water content, electrical conductivity, and snow frequency are the top predictors of biocrust presence and biomass. We predict that biocrusts are present in 2.7 × 10^5^ m^2^ and contain 11–72 Mg of aboveground carbon in the Lake Fryxell basin, often occurring on soils associated with seasonal snow patches. Our study illustrates the synergistic effect of combining field and remote sensing data for understanding and predicting ecological dynamics in the context of a changing climate.

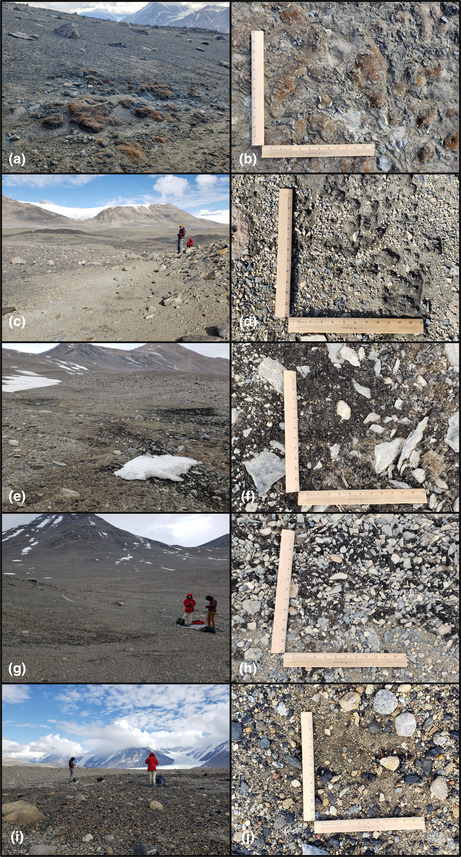

## 1 INTRODUCTION

Drylands, Earth's largest biome (Schimel, 2010), cover approximately 45% of the Earth's land surface (Prăvălie, 2016) and store about 32% of the global soil organic carbon (SOC) pool (Plaza et al., 2018). Among these dryland ecosystems are unique phototrophic communities growing on the surface soil. Biological soil crusts (i.e., biocrusts) are soil bioaggregates containing communities of cyanobacteria, algae, moss, and lichen (Weber et al., 2022), inhabit all continents (Belnap et al., 2016), cover 12% of the Earth's terrestrial surface (Rodriguez‐Caballero et al., 2018), and play foundational roles in the ecosystems where they occur, such as stabilizing soil, influencing soil hydrology, governing nutrient cycles, and supporting biodiversity hotspots (Belnap et al., 2016). Biocrusts are considered ecosystem engineers since they modulate the availability of resources and therefore modify, maintain, and create habitats for other terrestrial species (Barrera et al., 2022). Moreover, biocrusts are often the primary source of fixed carbon in some drylands (Elbert et al., 2012).

Drylands are particularly sensitive to climate change and are projected to become even drier (Allan & Douville, 2023; Feng & Zhang, 2015; Prăvălie, 2016). Climate change, especially changes in precipitation and soil disturbances, is predicted to decrease biocrust abundance by 25%–40% within the next 60 years (Finger‐Higgens et al., 2022; Rodriguez‐Caballero et al., 2018). Additionally, changes in weather and climate patterns have already caused vegetation shifts in drylands (Schlaepfer et al., 2017) and may have complex impacts on biocrust distribution and abundance, for example, decline in vascular vegetation sometimes creates more available surface area for biocrust expansion (Chen et al., 2020).

The polar desert environment of continental Antarctica also hosts biocrusts, primarily consisting of cyanobacteria, bryophytes, algae, and lichen (Büdel & Colesie, 2014; Colesie et al., 2014; Green & Broady, 2001). Although sporadic in occurrence, biocrusts develop where there is a regular water supply (e.g., from melting snow), as well as shelter and sunlight (Green & Broady, 2001; Kennedy, 1993). Biocrusts often occur in micro‐niches where snow drifts accumulate and persist – typically in shallow depressions, on the lee side of boulders, and within the microtopography of periglacial features (Cannone & Guglielmin, 2010; Green & Broady, 2001). Cyanobacteria‐dominated biocrusts occur in the wettest areas, and bryophyte‐dominated biocrusts are more common along the margins of consistent water sources, typically as *Bryum‐Hennediella heimii* communities, with drier mosses often colonized by lichens (Büdel & Colesie, 2014; Green & Broady, 2001; Schwarz et al., 1992). Antarctic biocrusts form sheltered microhabitats associated with diverse soil fauna, hosting nematodes, tardigrades, rotifers, and ciliates (Colesie et al., 2014; Green & Broady, 2001; Power, Salvatore, Sokol, et al., 2024). They have also been shown to increase soil fertility (Barrera et al., 2022). For example, some cyanobacteria taxa fix atmospheric nitrogen to usable nitrogen forms for other organisms (Kohler et al., 2018), and biocrusts are a source of leached organic matter into the soils as well (Colesie et al., 2014; Power, Salvatore, Sokol, et al., 2024). This is an essential function of Antarctic biocrusts in particular, since the terrestrial environment of continental Antarctica is overall nutrient poor with low diversity. Biocrusts also stabilize soils, particularly the stems and rhizoids of moss aggregate soil particles (Colesie et al., 2014), as has been extensively shown in non‐Antarctic deserts as well (Belnap & Büdel, 2016).

The McMurdo Dry Valleys are the largest ice‐free area on the Antarctic continent (Levy, 2013) and are one of Earth's coldest and driest deserts with mean annual temperatures between −15 and −30°C and less than 50 mm water equivalent of snowfall annually (Fountain et al., 2010; Obryk et al., 2020). The Lake Fryxell basin in eastern Taylor Valley hosts the most productive soils in the McMurdo Dry Valleys and supports patchily distributed biocrust communities across the terrestrial landscape (Barrett et al., 2007; Power, Salvatore, Sokol, et al., 2024). This region also has the greatest snow accumulation, highest relative humidity, and the shallowest ice‐cemented permafrost layers in the Taylor Valley, and therefore has higher soil moisture content (Bockheim et al., 2008; Doran et al., 2002; Myers et al., 2022; Obryk et al., 2020). Recent work suggests that melting of seasonal snow patches is the primary source of liquid water to biocrust communities outside the glacial flow paths of meltwater streams and endorheic lakes (Power, Salvatore, Sokol, et al., 2024; Thapa‐Magar et al., in review).

Similar to other dryland ecosystems, Antarctica's climate is changing, and its unique vegetation is already responding (Colesie et al., 2023; Robinson et al., 2003). Predictions for the outlook of Antarctica's vegetation are multifaceted, as vegetation responses to changes in temperature, water availability, and snow, for example, are complex and taxa‐specific, and as climate trends are different in maritime and continental Antarctica, likely leading to dissimilar vegetation responses among the two bioregions (Colesie et al., 2023). Increasingly dynamic weather and general climate warming are predicted for the McMurdo Dry Valleys region (Fountain et al., 2014; Nielsen et al., 2023; Obryk et al., 2020), and recent observations have illustrated significant responses of terrestrial and aquatic ecosystems to extreme weather events (Barrett et al., 2024; Gooseff et al., 2017). More variable climate and dynamic weather will likely influence the distribution and abundance of biocrusts in the McMurdo Dry Valleys in unknown ways. Therefore, understanding the controls of biocrust distribution is the essential first step in elucidating the impacts of climate change on the base of the McMurdo Dry Valleys soil ecosystem and organic C balance.

**FIGURE 1:**
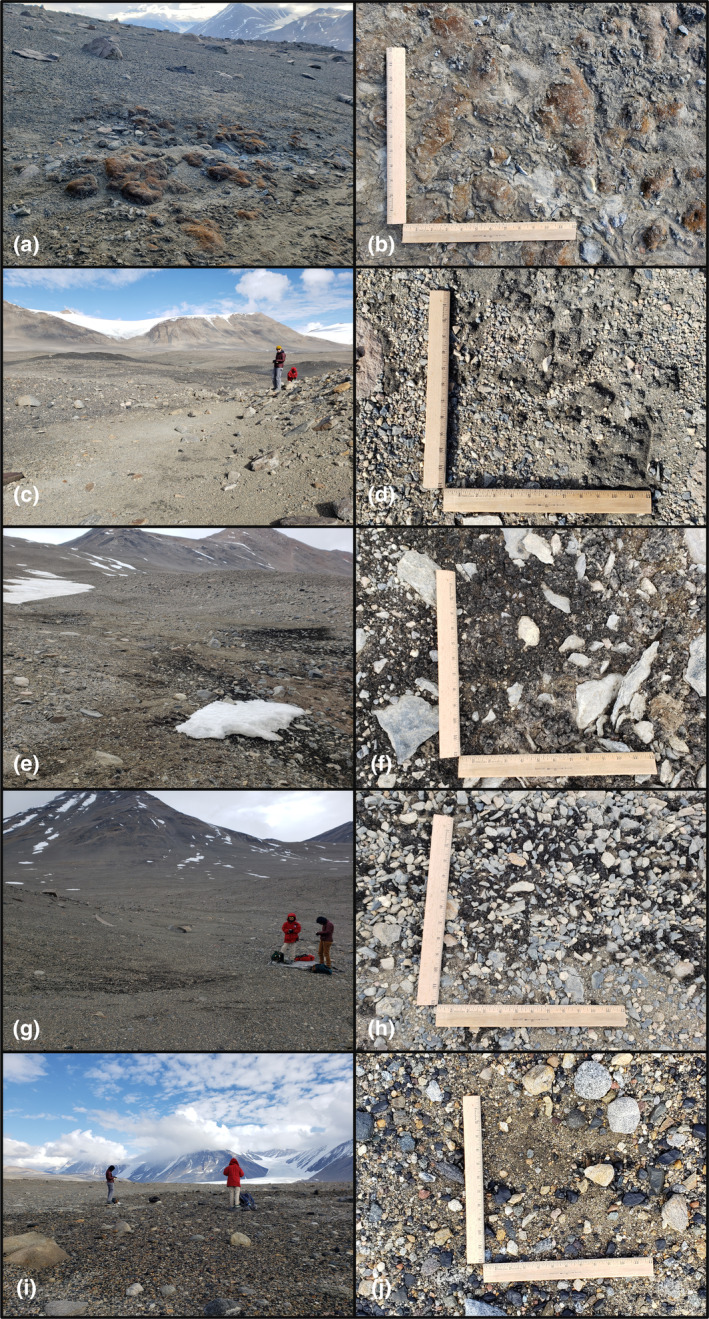
Photographs illustrating the composition and density of biocrust communities sampled. Among the represented communities are: (a–d) bryophyte‐dominated biocrusts, (e–h) cyanobacteria‐dominated biocrusts, and (i, j) incipient biocrusts. Landscape photographs illustrate the patchiness of the communities, and the close‐up photographs include 1 ft (~30 cm) scales. The following sampling sites are represented: (a, b) site 64, (c, d) site 32, (e, f) site 62, (g, h) site 61, and (i, j) site 36. Photographs taken by S. Power and J. Jorna.

Here, we describe the distribution and abundance of biocrust communities occupying soil environments in the Lake Fryxell basin outside of stream channel and lake margin environments (Figure 1). We used a combination of in‐situ field surveying and sampling, remote sensing, and machine learning algorithms to: (1) assess the main drivers of biocrust distribution and biomass, (2) model the distribution of biocrust presence and biomass, and (3) estimate the total surface organic C content of biocrusts across soils of the Lake Fryxell basin of Taylor Valley, Antarctica. This initial assessment is essential to ultimately characterize and monitor biocrust distribution and habitat suitability as a baseline against a changing regional climate.

## 2 MATERIALS AND METHODS

Sampling locations were identified in the Lake Fryxell basin using multispectral satellite data to select sites over a range of soil moisture contents and biocrust cover. These sites were visited, surveyed, and sampled, and physical and chemical characteristics of biocrust and surface soil were measured in the laboratory. Potential predictors of biocrust presence and biomass include these field survey‐based variables and geospatial raster variables which were extracted from each site using digital elevation models (DEMs) and multispectral satellite data. A series of random forest algorithms were developed to assess the top predictors of biocrust presence/absence and ash‐free dry mass (AFDM). Maps of biocrust presence and AFDM were created for the Lake Fryxell basin, and projections of aboveground organic C were estimated.

### 2.1 Field site selection

**FIGURE 2:**
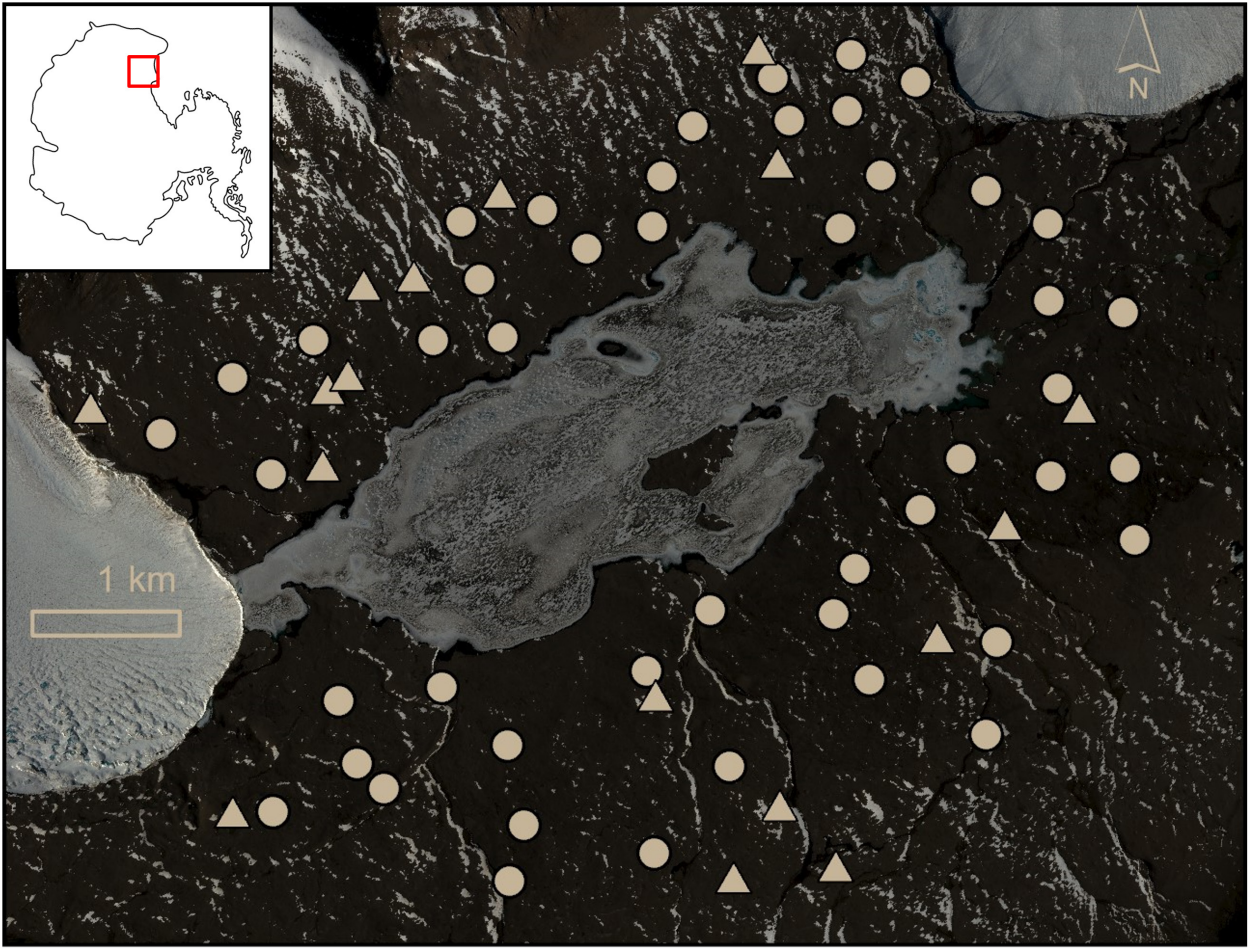
Map of Eastern Taylor Valley with 64 field sites labeled with triangles (biocrust present) and circles (biocrust absent). Inset on the top left identifies the McMurdo Dry Valleys, Antarctica with a red square. WorldView‐2 imagery (10300100CB9F3900), bands 5 (red), 3 (green), and 2 (blue) © Dec 21 2021 Maxar Technologies, Inc.

We identified 64 sites for ground truthing of satellite imagery and surveying and sampling of surface soils (Figure 2). These sites were selected to cover a range of predicted moisture content and predicted biocrust abundance using high spatial resolution WorldView‐2 and ‐3 multispectral satellite imagery (Maxar Technologies, Inc.). WorldView‐2 and ‐3 are in polar orbit with 8 multispectral bands in the visible and near‐infrared regions at 1.84 and 1.24 m spatial resolutions at nadir, respectively. The predicted moisture content model used was derived from albedo measurements relative to long‐term baseline surface albedos (Salvatore et al., 2023), and the predicted biocrust abundance model used was derived from spectral linear unmixing models (Power, Salvatore, Sokol, et al., 2024; Salvatore, 2015; Salvatore et al., 2020, 2021). The moisture content model used was a product of 21 WorldView‐2 and ‐3 images (see table A1, Salvatore et al., 2023), and the biocrust abundance model was based on the spectral unmixing of a WorldView‐3 image (10400100485D6900) acquired Jan 26, 2019. In ArcGIS Pro, we used conditional statements to identify pixels of a variety of conditions (i.e., wet, dry, barren, and/or abundant). We intentionally identified coordinates of areas covering a range of predicted moisture content and biocrust abundance and spatially spread throughout the Lake Fryxell basin (Figure 2).

### 2.2 In‐situ surveying and sampling

The 64 locations identified using multispectral satellite imagery, representing a range of predicted moisture content and biocrust abundance, were visited between December 20 to 25, 2022. Sites were photographed and surface features and conditions (e.g., snow patch presence, moisture status, etc.) were documented. In particular, the presence or absence of biocrust was documented based on visual observation and assessment using the contemporary definition presented in Weber et al. (2022). Sites were noted as having biocrust absent, incipient biocrust present (e.g., sparse or emerging colonies), or dense biocrust present. Additionally, we differentiated the biocrusts between bryophyte‐dominated and cyanobacteria‐dominated biocrusts (Figure 1), and noted the presence of lichen, hypoliths, or other photosynthetic communities near or part of the biocrusts. Incipient biocrusts were identified as areas with emerging colonies entrained within the sediment of the desert pavement surface. These incipient biocrusts appeared as cohesive cyanobacteria filaments either as part of dry and flaky surface layers or moist and firm connected surface layers. We considered biocrust as *present* at both dense sites where biomass covered large proportions of the survey areas as well as much sparser and patchier sites as well. Surface soil or biocrust was collected from bare desert pavement (128 cm^2^) or with a brass cork borer (2.27 cm^2^), respectively, at each site for subsequent pigment analysis (chlorophyll‐a, scytonemin, and carotenoids) and organic matter content as AFDM. Samples were collected from representative areas of the landscape. Underlying soil to 10 cm depth was also collected at each site for subsequent analysis of the gravimetric water content (GWC), pH, electrical conductivity (EC), inorganic nitrogen (N) and phosphorus (P) concentration, soil organic carbon (SOC), and total nitrogen (TN). Samples were kept frozen at −20°C in complete darkness until subsequent analyses were performed.

### 2.3 Laboratory analyses

We measured pigment concentration on the surface ~1 cm layer soil and biocrust samples using a trichromatic spectrophotometric method for chlorophyll‐a, carotenoids, and scytonemin at 663, 490, and 384 nm, respectively (Garcia‐Pichel & Castenholz, 1991). The dried samples were prepared by extracting the pigments in 90% unbuffered acetone for 24 h using a 3.75:10 soil to solvent ratio for samples which contained zero or minimal biocrust and a 0.5:10 biocrust to solvent ratio for samples containing dense biocrust. Details regarding the sample preparation, analysis using a spectrophotometer, and pigment calculation are provided in Power, Salvatore, Sokol, et al. (2024). Additionally, the surface layer soil and biocrust samples were measured for AFDM by weighing a known area of sample, oven drying, weighing, and then combusting at 550°C for 24 h using a muffle furnace, gently stirring samples halfway through combustion, and reweighing after cooling in a desiccator.

The pH and EC were measured in the underlying soil samples using a 1:2 and 1:5 soil to DI H_2_O slurry, respectively, and GWC was determined as the mass of water lost after oven drying at 105°C for 24 h. We also extracted inorganic N (NH_4_^+^ and NO_3_^−^) in 2 M potassium chloride and inorganic P (PO_4_^3−^) in 0.5 M sodium bicarbonate. Inorganic N and P were measured on extracts using a Lachat flow injection analyzer (Knepel, 2003; Prokopy, 1995). Additionally, we measured SOC and TN using an Elementar Vario MAX Cube analyzer after fumigating samples with concentrated hydrochloric acid to remove the influence of carbonates on SOC values (Walthert et al., 2010). SOC and TN were expressed as g C m^−2^ and g TN m^−2^ using a bulk density estimate of 1.6 g dry soil cm^−3^ (Levy & Schmidt, 2016) for the estimation of soil organic matter stocks (0–10 cm).

### 2.4 Habitat description and characterization

Since many of the physical and chemical properties of the soil were highly skewed (Figure S1), a Wilcoxon Rank Sum test was performed in R Statistical Software version 4.1.2 to assess differences between sites that contained biocrust and those that did not (R Core Team, 2021). Potential predictor variables of biocrust measured from samples collected in the field include both physical (GWC and EC) and chemical properties (pH, NH_4_^+^, NO_3_^−^, PO_4_^3−^, SOC, and TN). Other potential predictors of biocrust were selected using remotely derived datasets. These include aspect, slope, and elevation collected from a high resolution DEM (Fountain et al., 2017), a rasterized GWC layer developed for this region by Salvatore et al. (2023) using 21 WorldView‐2 and ‐3 images (see table A1, Salvatore et al., 2023), and a rasterized snow frequency layer also developed for this region using albedo thresholds with the same 21 images (Salvatore et al., 2023) over the course of the austral summer (see also Thapa‐Magar et al., in review) (Figure S2). Snow frequency was calculated as the percentage of the 21 WorldView‐2 and ‐3 images that indicate snow at each pixel relative to those images that do not indicate snow at each pixel (e.g., 0% representing no snow observed in any of the images analyzed and 100% representing snow observed in all the images analyzed).

### 2.5 Machine‐learning algorithm development

A series of classification and regression models were developed for predicting biocrust presence and AFDM using MATLAB's classification and regression learner with an out‐of‐bag (OOB) approach. Binary classification models were developed using biocrust presence and absence (based on visual survey) as the response variables and separately the field survey and rasterized geospatial variables as predictor variables. Regression models were also developed using AFDM as the response variable and using the same predictor variables as indicated above. MATLAB's classification learner tested 40+ different models (e.g., decision trees, support vector machines, nearest neighbors, Bayes classifiers, ensemble classifiers, neural networks) to assess whether our data could be successfully trained to classify data, as did MATLAB's regression learner (e.g., linear regression, regression trees, support vector machines, kernel approximation, ensembles of trees, neural networks), assessing whether our data could train regression models for prediction. Results from the classification and regression learner are presented as the Supplemental Text.

For our study, we chose to concentrate on the random forest algorithms and apply those to map predictions since these are widely used and understood in the species distribution modeling and geospatial communities (Cerrejón et al., 2020; Evans et al., 2011; Qiu et al., 2023). We ran a series of random forest models: (1) classification models for predicting biocrust presence and absence, and (2) regression models for predicting biocrust AFDM. These models were informed by “field survey” data, “geospatial raster” data, or “hybrid” approach that included both field and remotely sensed data. The quality of the models was evaluated using OOB predictions on the training data rather than setting aside test data; therefore, all data were used in training the models. All random forest models presented in this paper were developed using the R package ModelMap (Freeman & Frescino, 2009), which provides an interface between the randomForest (Liaw & Wiener, 2002) and PresenceAbsence packages (Freeman & Moisen, 2008), in R Statistical Software (v4.1.2; R Core Team, 2021).

### 2.6 Predicting biocrust presence/absence with random forest models

Presence or absence of biocrust observed at the 64 sites was the binary response variable, and the predictor variables were the field survey data measured for each site and the rasterized geospatial data calculated for each site as well, modeled separately and together (hybrid). The number of trees in the models were optimized based on the OOB error. The outputs included model accuracy, OOB error, area under the receiver operating characteristic curve (AUC), relative influence of the predictor variables, and confusion matrices which provided class errors. AUC scores range from 0 to 1 and are used to evaluate model performance; the closer the AUC score is to 1, the greater the prediction accuracy of the model (Elith et al., 2006; Hosmer & Lemeshow, 2000). The relative influence of the predictor variables is based on the mean decrease in accuracy, which expresses how much accuracy the model loses by excluding each variable, and the mean decrease in Gini, which is a measure of how each variable contributes to the homogeneity of the nodes and leaves in the model. These are both measures of variable importance; the variables with the greatest mean decrease in accuracy and greatest mean decrease in Gini are considered the most important variables in predicting biocrust presence/absence.

### 2.7 Predicting biocrust AFDM with random forest models

We chose to model AFDM, which provides information beyond whether a biocrust is present or not, but *how much* biomass is present, and can therefore be used to estimate carbon stocks. AFDM measured at the 64 sites was the continuous response variable in 2 random forest models using data from the field surveys and the rasterized geospatial data as the predictor variables. The number of trees in the models were optimized based on the OOB error. The outputs included measures of the relative influence of the predictor variables and observed versus predicted AFDM, which were used to calculate model *R* ^2^ and root mean square error (RMSE). The relative influence of the predictor variables is based on the percent increase in mean square error (MSE), which is a measure of the increase in error from randomly permuting each predictor, and the increase in node purity, which is based on changes in node purity from splitting on the variable. These are also both measures of variable importance; the variables with the greatest percent increase in MSE and greatest increase in node purity are considered the most important variables in predicting biocrust AFDM.

### 2.8 Mapping biocrust presence and AFDM

Maps of predicted biocrust presence were created using random forest models with geospatial predictors in the R package ModelMap (Freeman & Frescino, 2009). ModelMap provides an interface between the raster package to read and predict over raster data (Hijmans, 2023). Files of the detailed prediction surface images were created in R and opened in ArcGIS Pro for subsequent analysis and map production. Symbology thresholds for each map were created by presenting either a high likelihood of biocrust presence (>70%) or relatively dense AFDM locations (>150 g m^−2^). The resultant prediction map is the mean of all the trees, since the model for AFDM had a continuous response.

The presence/absence probability and AFDM models were used to estimate the area biocrusts are likely present within and basin‐wide aboveground C content in terrestrial environments of the Lake Fryxell basin, i.e., outside of stream channels and lake margins. Using ArcGIS Pro, the number of pixels containing a 90% probability of biocrust occurrence was determined and divided by the total number of pixels, providing a percentage of the Lake Fryxell basin that have biocrusts present. Multiplying the number of pixels that contain biocrusts with 90% certainty by the individual pixel area (~15 m^2^) resulted in the area of the Lake Fryxell basin that are predicted to have biocrusts present. The modeled AFDM of all pixels containing a 90% probability of biocrust occurrence was extracted from ArcGIS Pro. The range of these AFDM predictions was used to bracket a range of C (g C m^−2^) likely contained in biocrusts for the Lake Fryxell basin study area. Aboveground C content of this range was calculated as 53% of the AFDM, assuming organic matter is 53% C by weight (Wetzel, 1983). This AFDM range was then multiplied by the total area that biocrusts were present in (2.7 × 10^5^ m^2^).

## 3 RESULTS

### 3.1 Biocrust field surveys

Of the 64 sites visited, 17 contained visually conspicuous biocrust (Table 1), including dense or incipient morphologies, and 47 lacked visually discernible biocrust presence. The biocrusts identified and sampled were predominantly bryophyte‐dominated and cyanobacteria‐dominated biocrusts (Figure 1, Table 1). The bryophyte communities were primarily dark moss (likely *Bryum* spp.; Schwarz et al., 1992), and one site contained red moss (likely *Hennediella heimii*; Pannewitz et al., 2003). Sparse yellow and orange lichen were noted on some of the bryophyte‐dominated biocrusts. The cyanobacteria‐dominated biocrusts were predominantly black *Nostoc* spp. with active, green‐pigmented algae layers below the dark‐pigmented surface layer of some samples. The incipient biocrust communities contained emerging colonies of cohesive cyanobacteria filaments. Hypoliths were commonly found around the areas of biocrusts and incipient biocrusts, as previously identified in this region (e.g., Cowan et al., 2010). Many surveyed sites containing biocrust lacked snow cover at the time of sampling but had microtopographic features suggesting the presence of snow previously or earlier in the season (e.g., depressions, alluvial‐like features where snow melt moves surface soil downslope). These areas were confirmed with WorldView‐2 and ‐3 imagery to often contain early season snow cover in other seasons.

**TABLE 1:** Description of the 17 biocrust sites, including their location given by latitude and longitude in decimal degrees, organic matter content as AFDM, and short description of composition based upon field observation and visual assessment.

Site ID	Latitude	Longitude	AFDM (mg cm^−2^)	Dominant composition	Description
21	−77.60833	163.00967	2.9	Cyanobacteria	Incipient
23	−77.6116	163.07574	2.6	Cyanobacteria	Incipient
24	−77.60693	163.07674	23.8	Bryophyte	Sparse colonies
25	−77.60056	163.08643	4.4	Cyanobacteria	Incipient
26	−77.59479	163.12459	27.9	Bryophyte	Dark moss, orange lichen
32	−77.61436	163.26944	66.5	Bryophyte	Dark moss, nostoc
33	−77.62132	163.25098	13.6	Cyanobacteria	Light colored
35	−77.63176	163.20731	3.9	Cyanobacteria	Incipient, hypoliths
36	−77.6362	163.19463	8.6	Cyanobacteria	Incipient, filaments
37	−77.62522	163.17142	4.4	Cyanobacteria	Incipient
46	−77.58572	163.19687	49.4	Bryophyte	Dark moss, yellow/orange lichen
55	−77.63538	163.22357	27.9	Cyanobacteria	Nostoc
60	−77.6328	163.05199	4.5	Cyanobacteria	Light colored
61	−77.60608	163.0824	62.7	Cyanobacteria	Nostoc, dark moss
62	−77.59998	163.1006	44.5	Cyanobacteria	Nostoc
63	−77.59262	163.20291	62.3	Bryophyte	Dark moss, orange lichen
64	−77.60715	163.28993	77.3	Bryophyte	Red moss, nostoc

### 3.2 Soil physical and biochemical properties

Sites supporting biocrust had significantly higher concentrations of chlorophyll‐a, scytonemin, and carotenoid pigments in surface soils compared to sites lacking biocrust, with scytonemin the most abundant pigment among all sites (Table 2). Surface organic matter content in the form of AFDM ranged from 16.1 to 773 g m^−2^ and was highest in the biocrust sites and consistently lower in the sites lacking biocrust (Table 2). Soils supporting biocrust had higher moisture content, lower alkalinity, and lower EC compared to sites lacking biocrust (Table 2). Most of the soil variables had frequency distributions that were highly right‐skewed, roughly following power‐law frequency distributions, which are common in ecological data (Bak, 1996). EC data were particularly right‐skewed, due to several sites having very high EC, >5000 μS cm^‐1^ (Figure S1). For example, site 25, one of the sites supporting incipient biocrusts, had exceptionally high EC (~15,000 μS cm^‐1^), inorganic N, and among the highest SOC and TN values. While NH_4_^+^ and TN were not significantly different between sites containing biocrust and those lacking biocrust, SOC was significantly higher and PO_4_^3−^ and NO_3_^−^ were significantly lower in sites containing surface biocrust (Table 2). Two dense biocrust sites in particular had high SOC concentrations of 5.58 and 5.60 mg C g^−1^ dry soil (Figure S1), equating to ~900 g C m^−2^ in the surface 10 cm of soil.

**TABLE 2:** Median soil physical and biochemical properties and standard error of the median from each of the 64 sites (*n* = 17 biocrust present sites, *n* = 47 biocrust absent sites). GWC as the gravimetric water content, EC as electrical conductivity, AFDM as the ash‐free dry mass, SOC as soil organic carbon, and TN as total nitrogen. Wilcoxon rank sum *p*‐values shown based on the medians.

	Median of the biocrust present sites	Median of the biocrust absent sites	Wilcoxon rank sum *p*‐value
GWC (g g^‐1^)	0.08 ± 0.02	0.02 ± 0.01	<.0001
pH	8.24 ± 0.20	9.43 ± 0.13	<.001
EC (μS cm^−1^)	167 ± 1127	722 ± 403	<.01
AFDM (g m^−2^)	238 ± 80	24 ± 2	<.0001
Chlorophyll‐*a* (μg cm^−2^)	0.9 ± 1.0	0.0 ± 0.001	<.0001
Carotenoid (μg cm^−2^)	3.3 ± 1.4	0.03 ± 0.002	<.0001
Scytonemin (μg cm^−2^)	50.7 ± 39.1	0.4 ± 0.1	<.0001
NH_4_^+^ (μg N g^−1^ dry soil)	0.1 ± 0.08	0.1 ± 0.05	.67
NO_3_^−^ (μg N g^−1^ dry soil)	0.8 ± 38	6.3 ± 8	<.05
PO_4_^3−^ (μg P g^−1^ dry soil)	1.7 ± 0.6	2.8 ± 0.9	<.05
SOC (g C m^−2^)	147 ± 81	75 ± 7.7	<.01
TN (g TN m^−2^)	22 ± 14	21 ± 2.3	.33

### 3.3 Biocrust presence/absence and AFDM model performance

**FIGURE 3:**
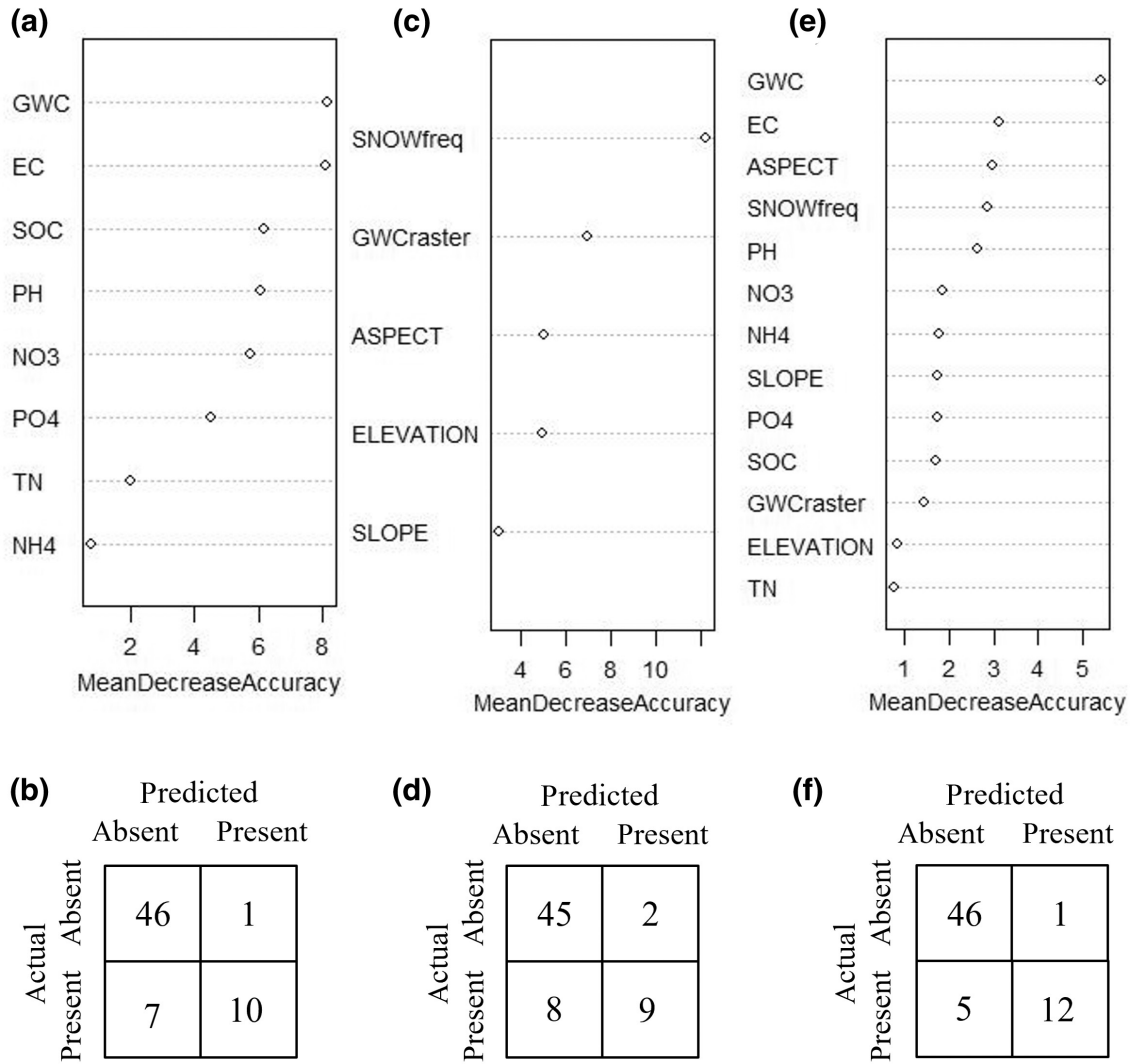
Variable importance (illustrated by mean decrease in accuracy) and confusion matrices for random forest models predicting biocrust presence/absence using (a, b.) field survey variables, (c, d) geospatial raster variables as predictors, and (e, f) both field survey and geospatial raster variables. The field survey model (a, b) had an overall accuracy of 87.5% and AUC of 0.86. The geospatial model (c, d) had an overall accuracy of 84% and AUC of 0.80. The hybrid model (e, f) had an overall accuracy of 91% and AUC of 0.89. Confusion matrices for the (b) field survey model, (d) geospatial raster model, and (f) hybrid model have class errors for absent and present as follows: 2.1 and 41%, 4.2 and 47%, and 2.1% and 29%.

The field survey model predicting biocrust presence using exclusively field survey derived variables had an overall accuracy of 87.5% (OOB error 12.5%), with class errors of 2.1% for absent and 41% for present (Figure 3b), and an AUC of 0.86. The confusion matrix indicates the field survey model underpredicts the presence of biocrust (Figure 3b). The GWC and EC were the most important variables when predicting biocrust presence in the field survey model, when comparing the mean decrease in accuracy (Figure 3a) and pH and EC were the most important when comparing the mean decrease in Gini (Figure S3a).

The geospatial raster model predicting biocrust presence using exclusively remote sensing derived variables performed with 84% accuracy (16% OOB error) and an AUC of 0.80. Snow frequency and rasterized GWC were the most important variables when predicting biocrust presence in the geospatial raster model, when comparing the mean decrease in accuracy and Gini (Figure 3c, Figure S3b). The class errors were 4.2% for absent and 47% for present, with the confusion matrix indicating the model underpredicts the presence of biocrust (Figure 3d).

A hybrid model predicting biocrust presence using both field survey and geospatial rasterized variables performed with the overall highest accuracy, 91% (OOB error of 9%), and AUC, 0.89. Field‐based GWC and EC were the most important variables when predicting biocrust presence in the hybrid model, followed by snow frequency, aspect, and pH according to the mean decrease in accuracy and Gini assessments (Figure 3e, Figure S3c). The class errors were 2.1% for absent and 29% for present (Figure 3f), indicating the hybrid model is more accurate when predicting areas where biocrust is absent, and it slightly underpredicts the presence of biocrust based on the confusion matrix.

**FIGURE 4:**
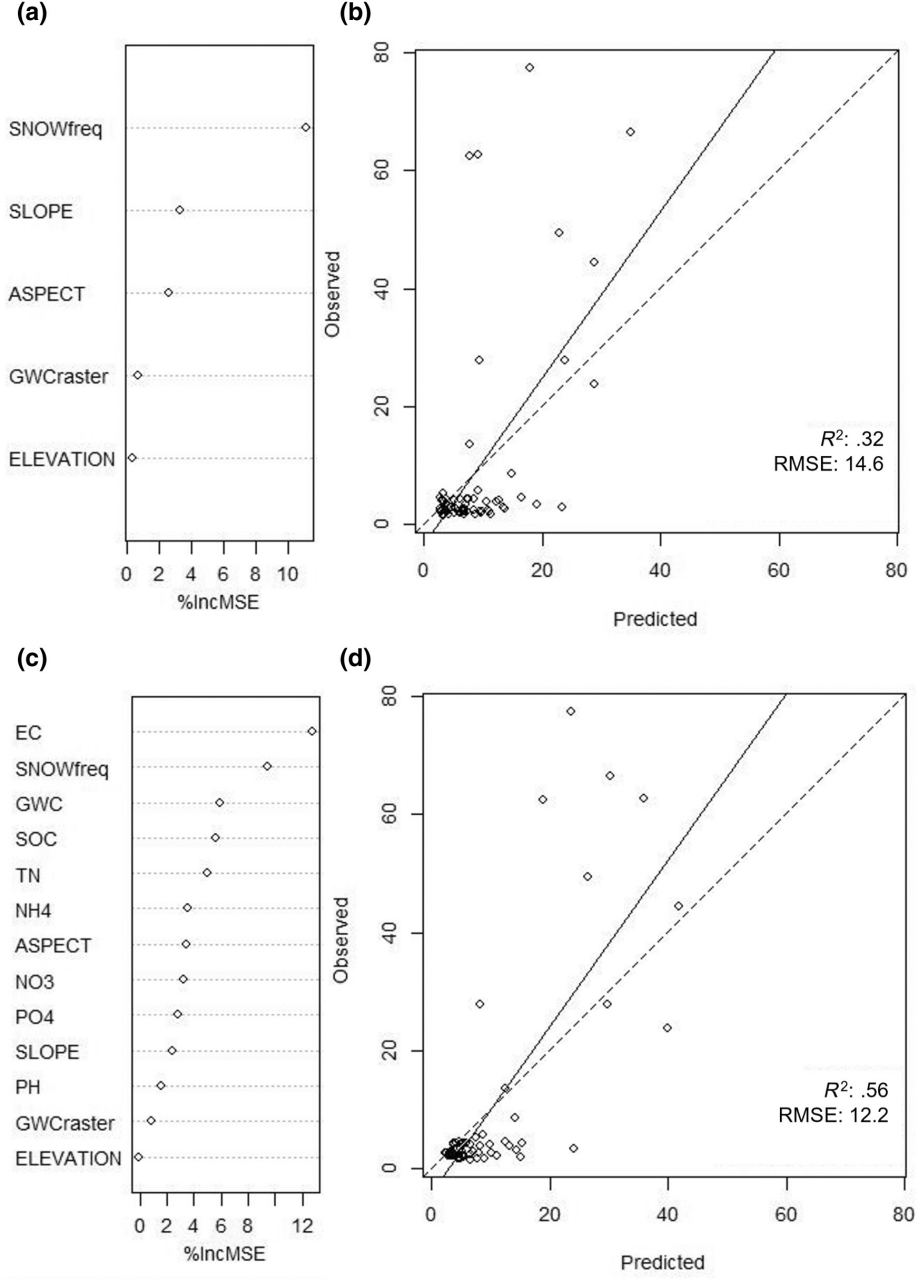
Variable importance (illustrated by percent increase in mean square error, MSE) and predicted versus observed plots for random forest models predicting biocrust AFDM using (a, b) geospatial raster variables and (c, d) both field survey and geospatial raster variables as predictors. The geospatial model (a, b) had an *R* ^2^ of .32 and an RMSE of 14.6. The hybrid model (c, d) had an *R* ^2^ of .56 and RMSE of 12.2.

The geospatial raster model predicting AFDM using exclusively remote sensing derived data had an *R* ^2^ of 0.32 and RMSE of 14.6, with a slight underprediction of AFDM based on the observed vs. predicted AFDM plot (Figure 4b). Snow frequency and slope were the most important variables when predicting AFDM in the geospatial raster model, when comparing the percent increase in MSE assessment (Figure 4a), and snow frequency and aspect were the most important variables when comparing the increase in node purity (Figure S4a).

The hybrid model predicting AFDM using both field survey and geospatial raster variables performed well with an *R* ^2^ of 0.56 and RMSE of 12.2, with a slight underprediction of AFDM based on the observed vs. predicted AFDM plot (Figure 4d). The most important variables predicting AFDM in the hybrid model were EC and snow frequency, when comparing the percent increase in MSE and node purity assessments (Figure 4c, Figure S4b).

### 3.4 Biocrust maps and carbon estimates

**FIGURE 5:**
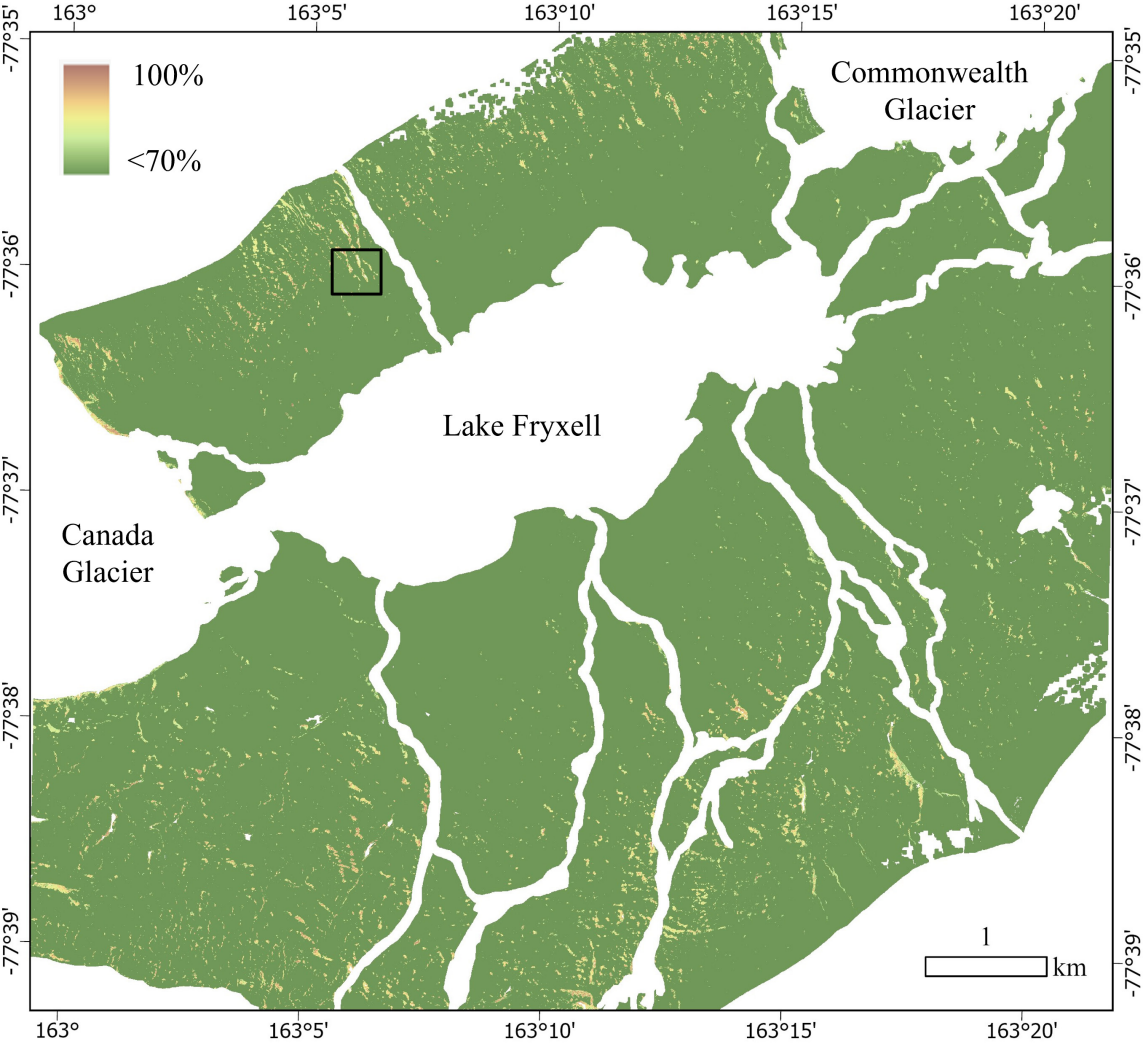
Probability surface map for presence of biocrust in the Lake Fryxell basin, Antarctica. The range of probabilities present here illustrates areas of highest (>70%) probability of biocrust occurrence. Areas shown in white are beyond the spatial limits of the input rasters or are areas masked out (glaciers, lake, streams, and perennial snow) to focus on the terrestrial landscape where biocrusts occur. Black square indicates location of Figure 6.

**FIGURE 6:**
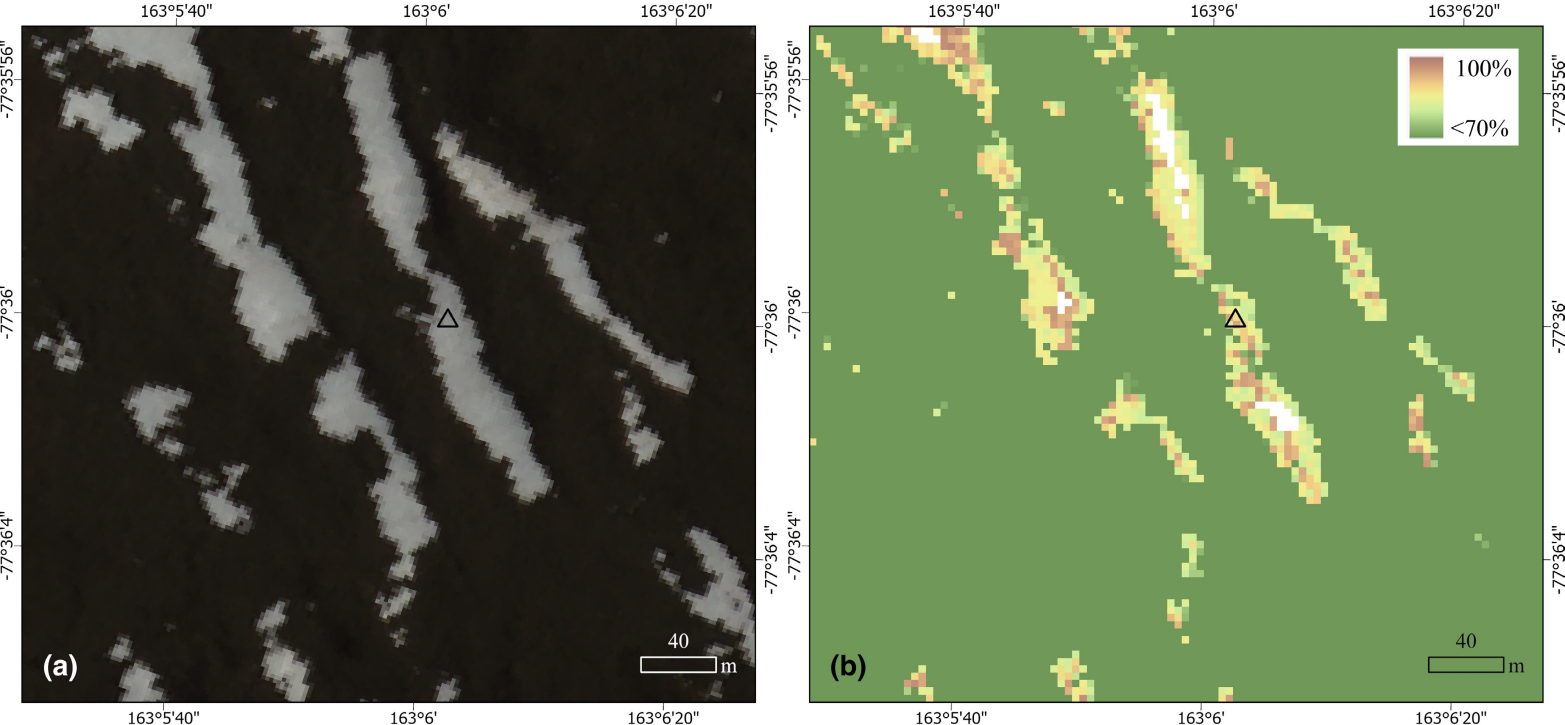
Two images of identical location in the Lake Fryxell basin, Antarctica. (a) Close up true‐color image showing seasonal snow patches, WorldView‐2 imagery (10300100CB9F3900) bands 5 (red), 3 (green), and 2 (blue) © Dec 212,021 Maxar Technologies, Inc., and (b) Close‐up image of the probability surface map for presence of biocrust. The range of probabilities present here illustrates areas of highest (>70%) probability of biocrust occurrence. Areas shown in white are pixels containing perennial snow cover which required exclusion. Black triangle denotes one of the sampling sites (site 62) containing dense biocrust cover located around snow patch features.

The predicted distribution of biocrust in the Lake Fryxell basin (Figure 5) at 90% probability occurred within 2.7 × 10^5^ m^2^. These areas of high probability of biocrust occurrence often coincided with areas of seasonal snow cover (e.g., Figure 6). The map of AFDM density follows a similar geographic pattern (Figure S5). Using both maps, we predict that areas with 90% probability of biocrust presence contain between 11 and 72 Mg of C in aboveground biocrust in 0.6% of the terrestrial Lake Fryxell basin landscape, excluding the masked‐out glaciers, lake, streams, and perennial snow areas.

## 4 DISCUSSION

Our models predict that biocrusts occur in 2.7 × 10^5^ m^2^ or in 0.6% of the arid terrestrial landscape of the Lake Fryxell basin (a conservative estimate based on 90% probability of presence), typically alongside and below seasonal snow patches (Figures 5 and 6). Physical and chemical analyses of the surface soils showed that biocrust sites are significantly different from unvegetated sites, especially for pigments and organic matter content (Table 2), illustrating the influence of biocrusts on soils. Field survey‐based data and remotely sensed data were both reliable predictors of biocrust presence and biomass, although hybrid models generated the most robust models. Remote sensing data are essential in informing our understanding of long‐term dynamics of snow (one of the most important predictors of biocrust occurrence and biomass) and are therefore essential in understanding the distribution of biocrusts and predicting their sensitivity to climate change. However, field sampling is an important complement to remote sensing efforts by highlighting important soil properties (e.g., EC) that are currently missing from our remote sensing capabilities. Our study illustrates the synergistic effect of combining field and remote sensing data for understanding and predicting ecological dynamics.

### 4.1 Primary drivers of biocrust distribution and biomass

The conditions that promote the densest microbial mats in the McMurdo Dry Valleys, for example, as found in the Canada Glacier Antarctic Specially Protected Area 131, are associated with wet to intermittently saturated soils that are often sheltered from foehn winds (Antarctic Treaty ASPA 131 Management Plan https://www.ats.aq/documents/recatt%5Catt683_e.pdf; Power et al., 2020). Here, we explicitly focused on the spatial distribution and drivers of lower density biocrust, which we found are also associated with moist conditions and sheltered within depressional features and snow patches. Random forest models provided robust, conservative predictions of biocrust presence and biomass (AFDM), i.e., they underpredict biocrust presence and biomass. All random forest classification models predicting biocrust presence performed with ‘excellent’ accuracy based on their AUC scores (Hosmer & Lemeshow, 2000), and the regression models predicting biocrust AFDM performed well with acceptable RMSE and *R* ^2^ values. While several variables were significant in our models, GWC, EC, and snow frequency were the strongest predictors of biocrusts, confirmed by two separate measures of evaluating variable importance per model (Figures 3 and 4, Figures S3 and S4). GWC is a more important variable in the models predicting biocrust presence (Figure 3) compared to those predicting biocrust AFDM, where EC and snow frequency were more important (Figure 4). Modeling AFDM provides information beyond whether a biocrust is present or not – it informs about the mass or density of the biocrust present. While GWC is important when predicting biocrust presence, we found that high frequency of snow cover and low EC are the habitat characteristics driving the densest biocrust sites (Figure 4).

We found that snow is an essential driver of biocrust presence and biomass in the Taylor Valley, evidenced by our random forest models, but also more simply by strong positive correlations between snow frequency and AFDM, chlorophyll‐a, and SOC (Table S1), confirming the importance snow presence has on biocrusts and the underlying soil C. In the McMurdo Dry Valleys, snow patches are documented to increase surrounding soil moisture compared to nearby snow‐free soils (Ayres et al., 2010; Eveland et al., 2012; Gooseff et al., 2003). The microtopography of periglacial features, shallow depressions, and the lee side of boulders collect snow drifts that accumulate in the winter and slowly melt in the summer months, sustaining biocrust communities (Cannone & Guglielmin, 2010; Green & Broady, 2001; Kappen et al., 1990). While dense and perennial snow covers have been shown to reduce the survival and growth of some lichen (e.g., “snowkill”; Sancho et al., 2017), the seasonal snow patches that slowly melt during the summer often sustain dense bryophyte‐ and cyanobacteria‐dominated biocrusts (Kappen et al., 1990; Power, Salvatore, Sokol, et al., 2024). Shallow layers of snow (~15 cm or less) allow sufficient light penetration for photosynthesis, and Antarctic vegetation below have been shown to remain active (Kappen & Breuer, 1991). Snow patches provide benefits beyond moisture availability, and therefore serve as preferential microhabitats for biocrusts and other organisms in Antarctica (Kappen, 1985; Power, Salvatore, Sokol, et al., 2024) and other ecosystems outside of Antarctica (Hao et al., 2020; Hui et al., 2022; Ladrón de Guevara & Maestre, 2022; Yang et al., 2023; Zhang et al., 2021). For example, snow cover reduces temperature extremes in underlying soil (Schimel et al., 2004), supplies nutrient inputs (Wright et al., 2024), and likely provides refuge for biocrusts from both eolian scouring during common extreme wind events (e.g., Beane, 2021; Nylen et al., 2004; Obryk et al., 2020) and intense UV‐radiation (e.g., Bernhard & Stierle, 2020; Farman et al., 1985; McKnight et al., 1999).

Water availability is essential, but it is not the sole environmental factor controlling the distribution and abundance of photosynthetic life in Antarctica (Kennedy, 1993). The interaction of water and soluble salts can create wet, salty sites that are inhospitable to biocrusts, likely because of the physiological stress associated with salts, as has been documented in the “water track” environments of the McMurdo Dry Valleys (Kuentz et al., 2022; Levy et al., 2011, 2014). For example, dense biocrust sites with the highest AFDM values (>400 g m^−2^) all had EC under 55 μS cm^−1^, while the average EC for sites without biocrust was 1700 μS cm^−1^. This interaction of salt and water is modulated by the topography and the timing of snow melt and permafrost thaw. For example, the frequency of melt events influences how often the soils and salts are hydrated, while topographic conditions influence whether those salts are flushed downhill. There is wide variation in GWC and EC, for example, within the sites containing biocrust and those not containing biocrust (Figure S1). Site 25 is an ecological outlier with low AFDM but high SOC, and exceptionally high EC (~15,000 μS cm^−1^) and nitrate concentrations. Several of our study sites that lacked biocrust had sufficiently high GWC (Figure S1); indicating that while water is essential for the development of biocrust, other factors can limit establishment and growth (e.g., exposure, insufficient nutrients, or excess salts). Other landscape factors like aspect and slope also contribute significantly to the random forest models predicting AFDM. For example, south facing slopes in the Lake Fryxell basin receive less intense solar radiation (Dana et al., 1998) and therefore likely have higher soil water content and greater seasonal snow accumulation.

Some of the top predictors for biocrust presence (i.e., snow and GWC) can be remotely measured via satellite imagery and, therefore, can be incorporated into models of landscape‐scale biocrust distribution. However, there are important field‐based geochemical variables, especially EC, that are not yet remotely measurable from satellite imagery and, therefore, could not be included in our geospatial‐based models. Weaker performance of the geospatial‐based models in comparison to the field survey‐based models is likely due to the absence of important edaphic variables. Future work incorporating geochemical data into these geospatial models will likely improve model performance (e.g., resistivity surveys using airborne instruments, Gutterman et al., 2023), and therefore improve accuracy into our prediction maps used for monitoring biocrust communities in a changing climate.

### 4.2 Contribution of biocrusts to carbon budget

Globally, biocrusts and other cryptogams (i.e., plants that have no true flowers or seeds) contain approximately 4.9 Pg of C or ~1% of the C content of terrestrial vegetation (Elbert et al., 2012), which includes other organisms beyond the contemporary definition of biocrusts (e.g., organisms growing in or on rocks, leaves, and wood) (Weber et al., 2022). This small fraction belies the local importance of biocrusts where they are sometimes the primary source of C in arid ecosystems. At a glance, the terrestrial landscape of the Taylor Valley appears barren and devoid of vegetation outside of the lakes and ephemeral streams which host dense microbial mats (Alger et al., 1997; Kohler et al., 2015; Stone et al., 2024). However, biocrusts occupy the soils in microhabitats suitable for their growth and contain a significant amount of C, influencing the organic matter content of the underlying soils as well (Power, Salvatore, Sokol, et al., 2024).

We estimate a range of biocrust biomass of 40–265 g C m^−2^ in pixels with a greater than 90% probability of biocrust occurrence, which scales to 11–72 Mg of aboveground biocrust C in the Lake Fryxell basin. Our models used to map biocrusts and AFDM likely underpredict the presence and biomass of biocrust but represent a first attempt at including these types of communities in regional C budgets. Scalable and systematic estimates of aboveground C are limited for this region, primarily due to the logistical challenges of conducting Antarctic field work. Early work by Burkins et al. (2001) estimated an average of 150 g C m^−2^ for the top 20 cm of soils in the Taylor Valley, in which they note that soils occupy over 95% of the non‐glacial landscape. We found that soils hosting biocrusts contained, on average, 228 g C m^−2^ in the top 10 cm and soils lacking biocrusts contained 85 g C m^−2^ on average, bracketing the SOC estimates in Burkins et al. (2001). When combining the aboveground biocrust C estimate of all areas in the Lake Fryxell basin predicted with 90% probability to contain biocrust (40–265 g C m^−2^) and the average belowground C from biocrust sites measured in‐situ (228 g C m^−2^), we estimate a total range of 268–493 g C m^−2^ in areas containing biocrust.

Remote sensing tools are essential in characterizing ecological processes at landscape scales. More recently with the advancements in remote sensing technologies and applications, remote assessments of aboveground biomass in the Lake Fryxell basin have been conducted (Power et al., 2020; Salvatore et al., 2021), primarily validated for use in the aquatic environments of dense microbial mats, while some have focused on characterizing the surrounding soil landscape (Power, Salvatore, Sokol, et al., 2024). Power et al. (2020) estimated C stocks of 21.7 Mg in the Canada Glacier Antarctic Specially Protected Area alone, which is considered one of the most diverse and productive environments in Taylor Valley (Schwarz et al., 1992). Salvatore et al. (2021) estimated 757 Mg of C in the Lake Fryxell basin, using a model derived primarily from observations from soils and sediments near stream channels and lake margins, possibly overestimating C when extrapolating out to the drier terrestrial landscapes. These recent estimates of aboveground biomass in the Lake Fryxell basin included dense microbial mats from aquatic landscapes and are therefore much greater than ours reported here, which were produced from models specifically designed for the drier terrestrial landscape. Our estimate of 11–72 Mg C in biocrusts occupying drier landscapes of the Lake Fryxell basin suggests that a significant portion of the valley‐wide C budget lies outside the conventionally studied aquatic flow‐paths.

### 4.3 The future of Antarctic biocrusts in a changing climate

Climate change is likely to tip arid ecosystems, and cold deserts in particular, out of stable states (Reed et al., 2016; Wall, 2007). The distribution of Antarctic biocrusts and their role on the local C cycle are sensitive to changes in local and regional climate (e.g., Cannone et al., 2021; Colesie et al., 2023; Convey & Peck, 2019; Robinson et al., 2003; Siegert et al., 2019). Specifically, future snow dynamics are particularly important in the survival and distribution of Antarctic vegetation (Colesie et al., 2023; Green et al., 2011; Tarca et al., 2022). Increases in precipitation from the occurrence of extreme snowfall events in coastal Antarctica (Turner et al., 2019) in contrast to regional drying trends in continental Antarctica (Robinson et al., 2018) illustrates how climate variability is region‐specific in the Antarctic and is nonetheless anticipated to impact vegetation in varying ways (Colesie et al., 2023). There is strong interannual variability of snow accumulation and snow persistence in the McMurdo Dry Valleys (Myers et al., 2022), an increasingly dynamic region on the precipice of change (Colesie et al., 2023; Gooseff et al., 2017; Levy et al., 2018; Obryk et al., 2020; Wall, 2007). For example, in the McMurdo Dry Valleys, recent climate variation has included an extended cooling period (Doran et al., 2002; Obryk et al., 2020), summer floods (Gooseff et al., 2017), the first records of rainfall in Taylor Valley, and an autumnal heatwave (Barrett et al., 2024) which are likely to influence the distribution and activity of biocrust communities (Colesie et al., 2023). Antarctic coastal regions are also predicted to experience more frequent and intense rainfall by the end of the century (Vignon et al., 2021). These changing weather and climate patterns could have drastic implications for the distribution and abundance of biocrust communities. Predicting how increasingly dynamic weather and future climate will impact terrestrial ecosystems and carbon balance in terrestrial Antarctica will require this improved understanding of the environmental drivers of soil biocrust communities.

## AUTHOR CONTRIBUTIONS

**Sarah N. Power:** Conceptualization (equal); data curation (lead); formal analysis (lead); investigation (lead); methodology (equal); project administration (lead); software (lead); supervision (equal); visualization (lead); writing – original draft (lead); writing – review and editing (lead). **Valerie A. Thomas:** Formal analysis (supporting); methodology (supporting); resources (equal); software (supporting); writing – review and editing (supporting). **Mark R. Salvatore:** Conceptualization (equal); funding acquisition (equal); resources (equal); writing – review and editing (supporting). **J. E. Barrett:** Conceptualization (equal); formal analysis (supporting); funding acquisition (equal); methodology (equal); project administration (supporting); resources (lead); supervision (equal); writing – original draft (supporting); writing – review and editing (supporting).

## FUNDING INFORMATION

Funding for this work was provided by several awards from the National Science Foundation: #OPP‐1637708 and most recently #OPP‐2224760 for Long‐Term Ecological Research, and #OPP‐2046260 to M. Salvatore.

## CONFLICT OF INTEREST STATEMENT

The authors declare that they have no competing interests.

## Supporting information


Data S1.

## Data Availability

All data presented in this study are archived in the Environmental Data Initiative (EDI) Repository (Power, Salvatore, Adams, & Barrett, 2024).
