# Supplementary material for: Habitat suitability of biocrust communities in a cold desert ecosystem
Source: Ecol Evol. 2024 Jun 28;14(7):e11649. doi: 10.1002/ece3.11649 (PMC11213821; doi:10.1002/ece3.11649)
Supplement: Supplementary file 1 — Data S1. [file ECE3-14-e11649-s001.docx]

**Supplemental Text**

*Regression and classification learner performance*

Our field survey data and their residuals were not normally distributed and therefore we chose a nonparametric modeling approach rather than a parametric approach. When using MATLAB’s classification learner testing 40+ models, all had accuracies > 70%, with several ~ 90%, for both models containing the field survey predictors and the geospatial predictors. The 40+ models tested using MATLAB’s regression learner performed adequately well (R^2^ of ~ 0.70 for many models using the field survey predictors; R^2^ consistently lower for models using geospatial predictors). In both cases, the random forest algorithms performed significantly well, and even though there were some models that performed better than random forest, we focused on the random forest algorithms for this study. We also chose to implement an OOB hold-out which inherently lowers accuracies. Without using an OOB technique, our model accuracies were much higher, though we have chosen to incorporate it as a way to measure prediction error.

**Supplemental Tables & Figures**

**
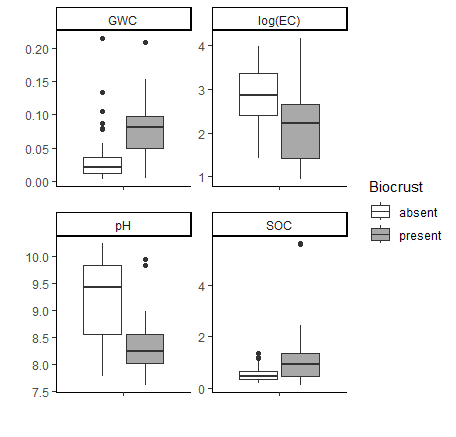
**

**Figure S1.** Boxplots of key soil characteristics where EC is electrical conductivity in μS cm^-1^, GWC is gravimetric water content in g/g, and SOC is soil organic carbon in mg C g^-1^ dry soil. Separate box plots are illustrated for plots where biocrust is absent (white) and where biocrust is present (grey). Horizontal lines indicate the median, and the black circles indicate outliers defined by > 1.5 x interquartile range (IQR). The whiskers represent the largest value within 1.5 x IQR above the 75th percentile and the smallest value within 1.5 x IQR below the 25th percentile.


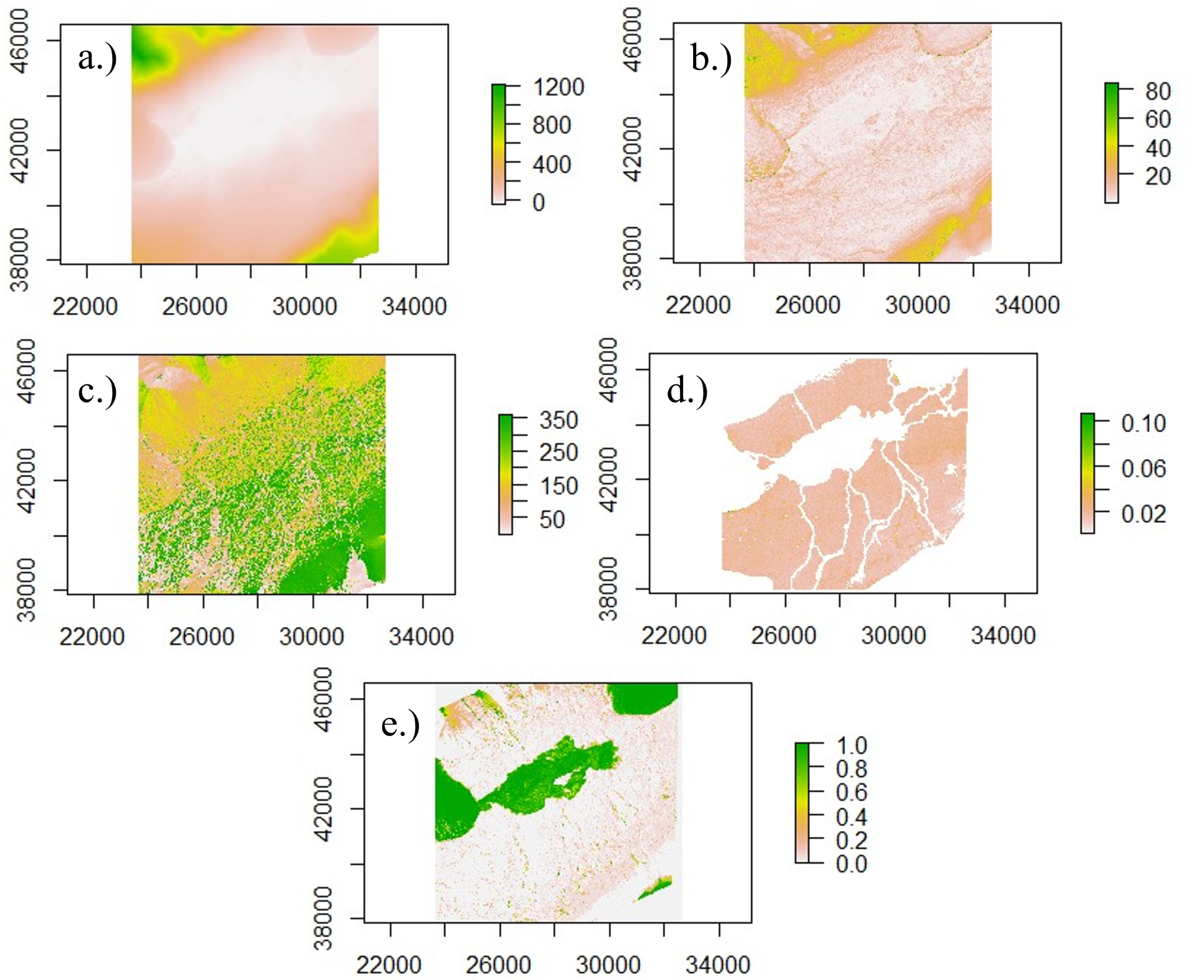


**Figure S2**. Raster images of the geospatial predictors: **a.)** elevation, **b.)** slope, **c.)** aspect, **d.)** gravimetric water content, **e.)** snow frequency.

**
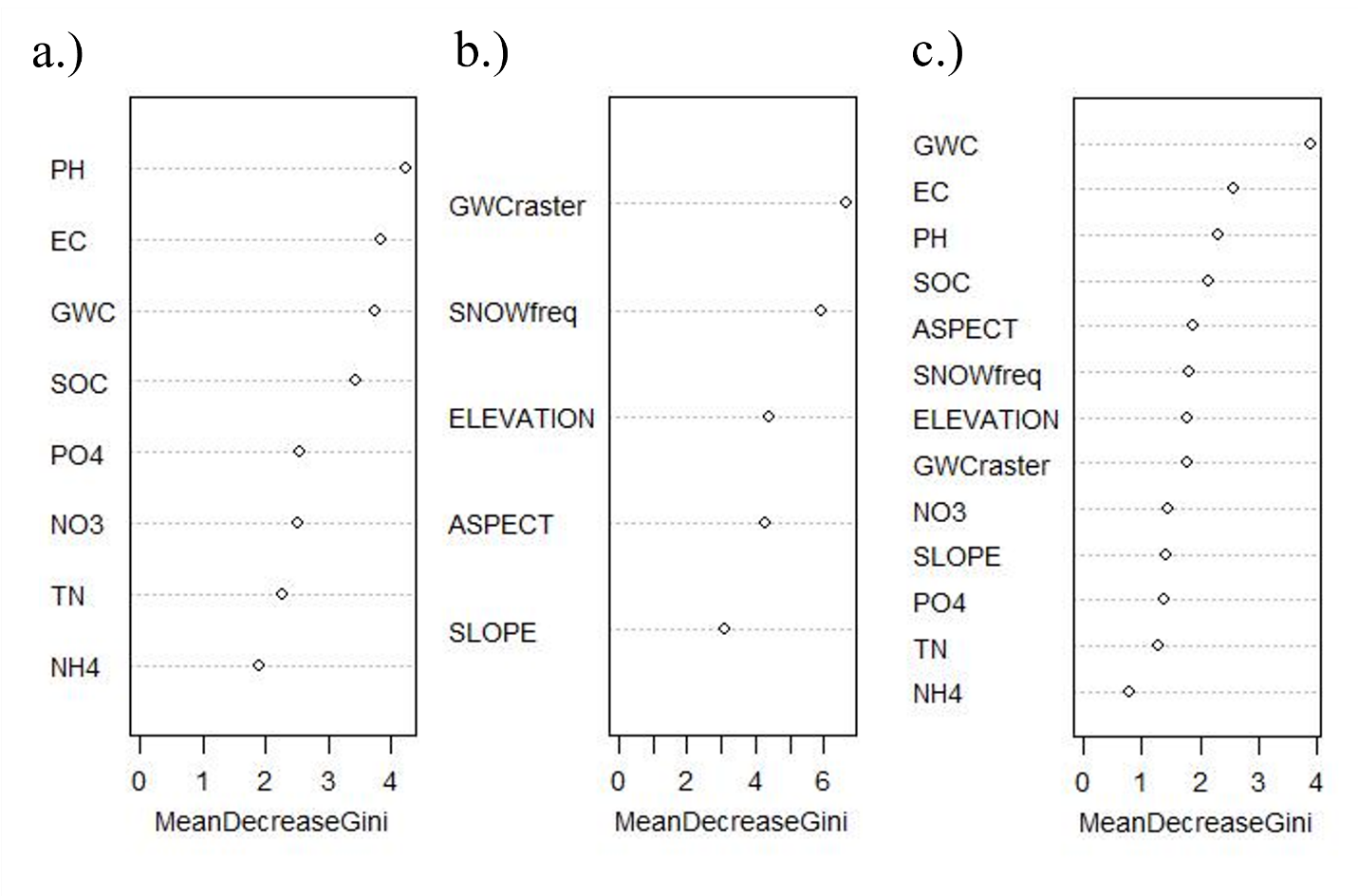
**

**Figure S3.** Variable importance (illustrated by mean decrease in Gini) for random forest models predicting biocrust presence/absence using **a.)** field survey variables, **b.)** geospatial raster variables, and **c.)** both field survey and geospatial raster variables as predictors.

**
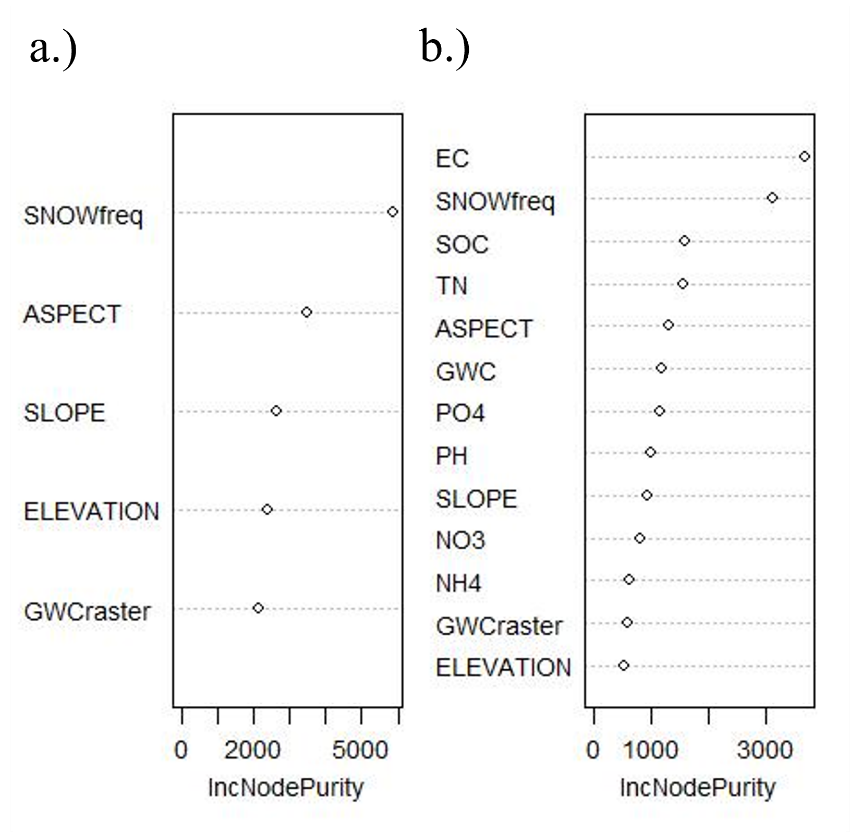
**

**Figure S4.** Variable importance (illustrated by increase in node purity) for random forest models predicting biocrust AFDM using **a.)** geospatial raster variables and **b.)** both field survey and geospatial raster variables as predictors.

**
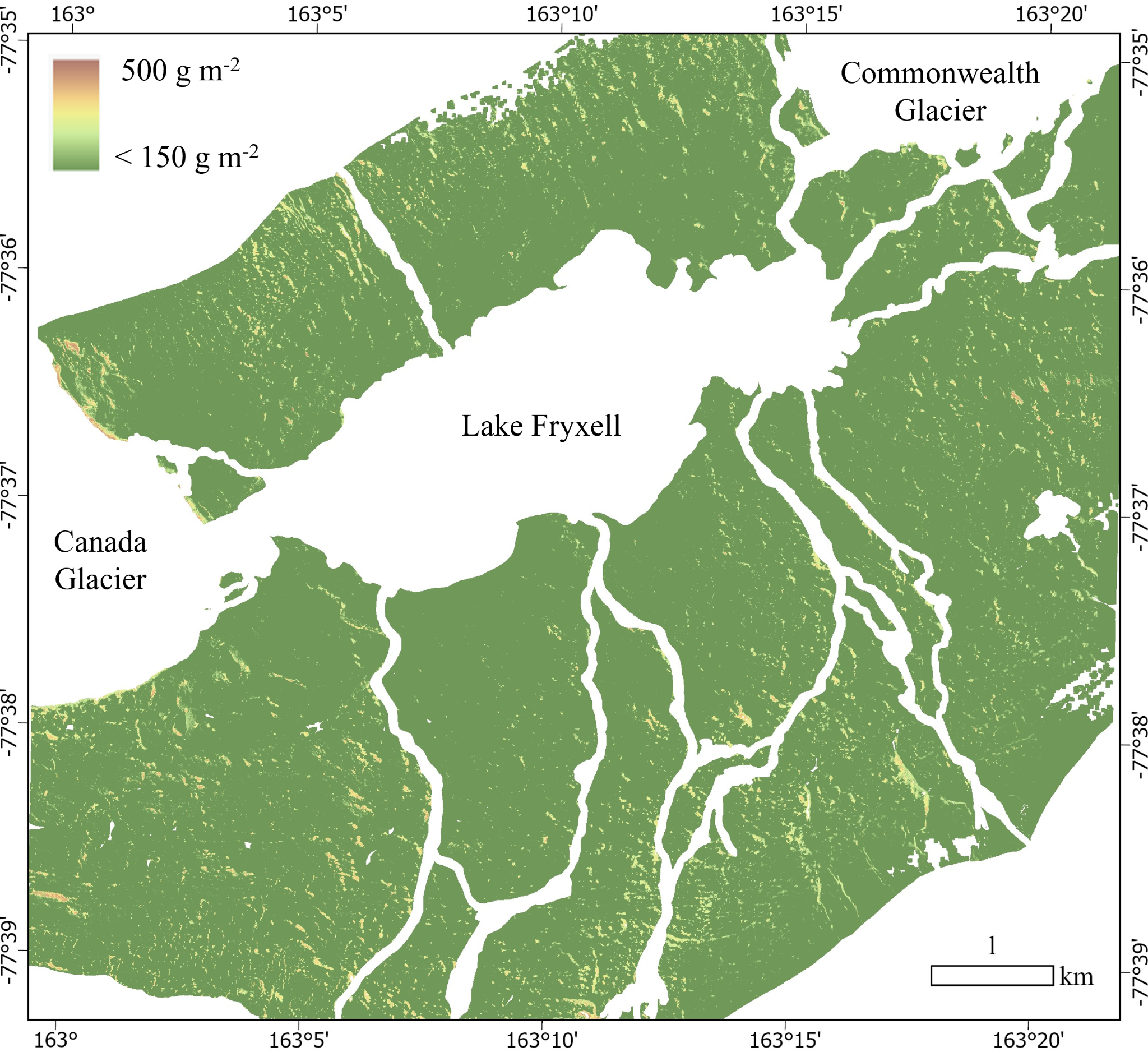
**

**Figure S5.** Predictive map of biocrust AFDM in the Lake Fryxell basin, Antarctica. The range of AFDM present here illustrates areas of relatively high AFDM (> 150 g m^-2^) density. Areas shown in white are beyond the spatial limits of the input rasters or are areas masked out (glaciers, lake, streams, and perennial snow) to focus on the terrestrial landscape where biocrusts occur.

**Table S1.** Correlation matrix using Pearson correlation coefficient (r) for all variables collected and tested in this study. All significant r values magnitude > 0.6 are indicated in bold.
